# Colon Adenocarcinoma during Pregnancy: A Case Report and Review of the Literature

**DOI:** 10.1155/2020/8894722

**Published:** 2020-11-22

**Authors:** Paolo Petruzzelli, Roberto Zizzo, Elisabetta Tavassoli, Miriam Sutera, Michela Chiadò Fiorio Tin, Luca Petruzzelli, Mariachiara Benedetto, Maria Rosa D'Anna, Paolo De Paolis, Guido Menato

**Affiliations:** ^1^Gynecology and Obstetrics, Department of Surgical Sciences, City of Health and Science, University of Turin, Turin, Italy; ^2^Department of Emergency Surgery, City of Health and Science, Turin, Italy; ^3^Department of General and Specialistic Surgery, City of Health and Science, Turin, Italy

## Abstract

Colorectal cancer (CRC) during pregnancy presents an estimated incidence of 1 : 13,000, and it is associated with diagnostic and therapeutic challenges. Here, we present the case of a 38-year-old woman, 25 weeks and 5 days pregnant, who was transferred to our Obstetrics and Gynecology Department from a local hospital with the diagnosis of intestinal obstruction. Magnetic Resonance Imaging (MRI) showed marked distension with hydroaerial levels of the enterocolic loops upstream of a concentric parietal thickening of the descending colon, stenosing, extended longitudinally for about 4 cm. An exploratory laparotomy was performed with resection of the colon splenic flexure and mechanical end-terminal anastomosis. Histological examination of the operating piece highlighted the presence of moderately differentiated (G2) colon adenocarcinoma (stage pT3N1b). The operation was followed by a single course of oxaliplatin and 5-FU plus leukovorin. The patient had a vaginal delivery at 37 weeks + 2 days of gestational age, following induction of labor and giving birth to a male infant whose weight was 2670 gr with apgar 9/9. We underline the pivotal role of attention to unspecific symptoms, early diagnosis, and active treatment in changing the clinical course of CRC.

## 1. Introduction

The incidence of malignancy during pregnancy is estimated at 1 : 1000 pregnancies, with an estimated incidence of colorectal cancer (CRC) of 1 : 13,000 [1]. Because of the fact that CRC signs and symptoms, including abdominal pain, nausea, vomiting, and altered bowel movements, are generally found even in normal pregnancy, the diagnosis may be late [2–5]. A diagnosis of CRC during pregnancy imposes therapeutic challenges in addition to major psychosocial issues. Here, we present a case of colon adenocarcinoma with diagnosis, surgery, and chemotherapy occurring during pregnancy.

## 2. Case Report

A 38-year-old woman (para 1001) presented at our Obstetrics and Gynecology Department: she was 25 weeks and 5 days pregnant with a 1-week history of absolute constipation, epigastric pain, vomiting, and distension. She had been transferred from a local hospital with the diagnosis of intestinal obstruction made by ultrasound and X-ray: there, she was subjected to RDS prophylaxis and tocolytic therapy with atosiban. Her pregnancy had been developing normally, and she had a previous vaginal delivery. Her family history was negative for cancer.

Obstetric examination showed no abnormalities. Initial laboratory results included a mildly elevated white cell count, PCR 22.7 mg/L, hemoglobin 10.6 g/dL, and a mean corpuscular hemoglobin 26.8 pg. Her serum potassium was 3.5 mmol/L. Liver function tests showed that the serum albumin was 3.4 g/dL. Urinalysis was normal.

**Figure 1:**
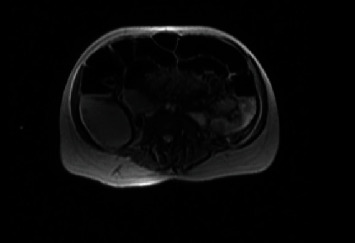
Magnetic Resonance Imaging.

A surgery specialist was consulted, and an abdominal Magnetic Resonance Imaging (MRI) was prescribed. The MRI showed marked distension with hydroaerial levels of the enterocolic loops upstream of a concentric parietal thickening of the descending colon, stenosing, extended longitudinally for about 4 cm (Figure 1); some lymph nodes with less than one cm diameter in the adjacent perivisceral adipose tissue were also reported as well as a perihepatic, perisplenic fluid stratum which was also present in the pelvic excavation.

Because of the clinical picture, in agreement with the patient, it was decided for exploratory laparotomy with resection of the colon splenic flexure and mechanical end-terminal anastomosis. Histological examination of the operating piece highlighted the presence of moderately differentiated (G2) colon adenocarcinoma (stage pT3N1b) infiltrating the subserous adipose tissue with the presence of lymph node metastases (2/14). Neoplastic cells were absent in the examined free fluid. The postoperative course was uneventful with late canalization (on the sixth postoperative day).

Postoperatively, the patient was in a good condition without fever and bleeding: she was discharged with medical and hygiene instructions.

The operation was followed by a single course of oxaliplatin and 5-FU plus leukovorin taking place in another hospital center.

Thirty-three days after surgery, the patient presented with abdominal pain in the upper quadrants, and she was hospitalized in our ward again: laboratory tests and obstetric ultrasounds showed no abnormalities, and she was discharged 4 days later in good general conditions with a booked check-in in the Day Service clinic.

Subsequent medical examinations showed a regular evolution of pregnancy; therefore, the patient was hospitalized again at 37 weeks of gestational age for induction of labor by means of endovaginal prostaglandins followed by amniorrhexis and infusion of intravenous oxytocin. Epidural analgesia was administered at the patient's request. Laboratory tests and cardiotocography were normal. She had a vaginal delivery at 37 weeks + 2 days of gestational age, giving birth to a male infant whose weight was 2670 gr with apgar 9/9, and she presented a first-grade perineal laceration which was promptly sutured. The total blood loss estimated at delivery was 300 mL. Considering the ongoing chemotherapy, it was decided to inhibit lactation with cabergoline. The patient was discharged on the third day after delivery with antithrombotic prophylaxis.

## 3. Discussion

Malignancy during pregnancy has recently become a major cause of maternal death in developed countries. The incidence of malignancies coinciding with pregnancy increased from 1 : 2000 in 1964 to 1 : 1000 deliveries in 2000 [1, 6, 7]. The increase is attributed to not only higher rates of cancer in general but also delays in childbearing to the third and fourth decades of life for women [4, 6].

Colon cancer is the third most common cause of mortality from cancer in women [8]; some cases are related to inherited susceptibility, but most of them are sporadic cancers [9].

The incidence of CRC is increasing in people < 50 years old [10]. Only 21% of all patients diagnosed with CRC are diagnosed before 55 years of age [10].

Our patient was 38 years old and did not have any risk factors for CRC.

Early-onset CRC more often presents at a more advanced stage and with more aggressive histologies [9, 11, 12].

The reported incidence of CRC in pregnant women is 1 : 13,000 [1]. Its symptoms typically include nausea, vomiting, abdominal pain, and altered bowel movements, which commonly overlap with symptoms of normal pregnancy [2–4]. Early diagnosis of CRC during pregnancy is difficult because the disease does not present specific symptoms and the presenting symptoms are associated with normal physiologic changes during pregnancy, until an obstruction or perforation happens [13].

Our patient had experienced abdominal pain, nausea, vomiting, and constipation at 25 weeks of gestation, and until that moment, she had not showed any symptoms.

A majority of CRC cases in pregnancy present with Duck class C (44%) in which adjutant therapies are needed to improve the surgical outcome [4, 14].

Although CRC during pregnancy is often diagnosed at a more advanced stage, patient survival has been reported to be comparable with that in nonpregnant patients [9, 15].

There has been much debate on the use of diagnostic imaging during pregnancy [16]. Ultrasound and MRI are the preferred forms of imaging in pregnancy, as they do not expose the fetus to ionizing radiation [17, 18]. Ultrasound remains the primary choice in the diagnosis of abdominal disease, but sensitivity for CRC is low, and further diagnostics are frequently necessary when ultrasound is negative [19]. MRI without the use of gadolinium has been proven useful in the diagnosis of abdominal problems during pregnancy and should be the next diagnostic tool when available [17, 20–22]. In fact, ACOG states that gadolinium use should be limited in pregnancy but may be used if it significantly improves diagnostic performance and is expected to improve fetal or maternal outcomes [23].

When MRI is unavailable or inconclusive, Computed Tomography (CT) can be of great value. However, especially during the first two trimesters of pregnancy, CT should be used with caution due to the uncertain effects on the fetus [17, 22, 24].

Moreover, pregnancy is considered a relative contraindication to colonoscopy due to risks of placental abruption from mechanical pressure, exposure to possible teratogenic medications, and fetal injury resulting from maternal hypoxia or hypotension during the procedure [17, 25].

In this case, MRI has been a pivotal tool for diagnosis while colonoscopy was not performed in order to avoid the risk of fetal injury.

When persistent bowel obstruction is diagnosed or highly anticipated, exploratory laparotomy must be commenced since prompt operative intervention maximizes the outcome for both fetus and mother [17].

Adjuvant chemotherapy for advanced CRC stages improves overall survival when started within six to eight weeks of surgery. This survival benefit declines significantly if adjuvant chemotherapy is delayed beyond three months [17, 19]. The most commonly used agent for CRC is 5-fluorouracil with iritocetan or oxaliplatin; however, more data exist for 5-FU during pregnancy [17, 26]. In our case, chemotherapy with oxaliplatin and 5-FU plus leucovorin was prescribed.

In the literature, there are two pivotal series that review CRC during pregnancy. The first one was published by Pellino et al. [18] in 2017 and included 79 papers reporting on 119 patients with an average age at neoplasm diagnosis of 32 years (range: 17–46). Diagnosis was made during the second and third trimesters in 41 and 47%, respectively. Obstruction occurred in 9.4% of patients while the most common presenting symptom was bleeding (47%) followed by abdominal pain (37.6%). Localization of the tumor was the colon in 53.4% of cases, the rectum in 44%, and multiple sites in 2.6%. Eight (9.8%) patients out of 82 patients whose treatment was described received chemotherapy during pregnancy, and none of the newborns developed permanent disability, except for one case of hypothyroidism. Colonic resection, with or without fecal diversion, was performed in 32.9% of patients, and in 33.3% of these cases, it was preceded by vaginal delivery or cesarean section. Vaginal delivery occurred in 60% of cases. The mothers' average survival was 36 months (range: 0–360), and 72% of newborns were alive; patients with rectal cancer had longer survival compared with patients with colon cancer (*p* = 0.0072).

The second series was published by Kocián and de Haan [27] in 2019: data were collected from the International Network on Cancer, Infertility and Pregnancy series, and 41 patients were included (27 colon cancer and 14 rectal cancer). Advanced disease was present in 30 patients (73.2%). The median age at diagnosis was 32 years (range: 24–43). Nonacute clinical presentation was noted in 31 patients. A diagnosis was made in the second trimester in 46% of cases, and it was obtained by imaging in 9.8%, surgery in 24.4%, and endoscopy in 65.9%. The tumor was located in the colon in 27 patients (65.9%). During pregnancy, 21 patients (51.2%) received surgery and 12 patients (29.3%) received chemotherapy. Thirty-three patients (80.5%) delivered live babies (21 cesarean sections and 12 vaginal deliveries), and 26 children (78.8%) were born preterm. The one-year overall survival rate was 78.1%, and the two-year survival rate was 64.4%.

Our patient had a vaginal delivery at term without complications.

## 4. Conclusion

CRC in pregnancy is associated with diagnostic and therapeutic challenges. Bowel obstruction is difficult to diagnose as the signs and symptoms such as abdominal pain, distention, vomiting, and constipation can be easily attributed to normal pregnancy leading to late diagnosis in advanced stages. Moreover, pregnancy requires alternative use of diagnostic tools, further hampering rapid and appropriate diagnosis. We would like to highlight the importance of using diagnostic imaging promptly upon suspicion of serious abdominal pathology, and, as far as we know, when severe pathology such as bowel obstruction is diagnosed or highly anticipated, surgery should not be delayed. In conclusion, attention to unspecific symptoms, early diagnosis, and active treatment might be fundamental to change the clinical course of CRC.

## Data Availability

Data will not be shared to preserve medical confidentiality.
